# Anatomy of avian rictal bristles in Caprimulgiformes reveals reduced tactile function in open‐habitat, partially diurnal foraging species

**DOI:** 10.1111/joa.13188

**Published:** 2020-03-23

**Authors:** Mariane G. Delaunay, Carl Larsen, Huw Lloyd, Matthew Sullivan, Robyn A. Grant

**Affiliations:** ^1^ Department of Natural Sciences Manchester Metropolitan University Manchester UK; ^2^ School of Life Sciences University of Liverpool Liverpool UK

**Keywords:** feather morphology, feeding ecology, mechanoreceptors, nightjars, sensing

## Abstract

Avian rictal bristles are present in many species of birds, especially in nocturnal species. Rictal bristles occur along the upper beak and are morphologically similar to mammalian whiskers. Mammalian whiskers are important tactile sensors, guiding locomotion, foraging and social interactions, and have a well‐characterised anatomy. However, it is not yet known whether avian rictal bristles have a sensory function, and their morphology, anatomy and function have also not been described in many species. Our study compares bristle morphology, follicle anatomy and their association with foraging traits, across 12 Caprimulgiform species. Rictal bristle morphology and follicle anatomy were diverse across the 12 species. Nine of the 12 species had mechanoreceptors around their bristle follicles; however, there was large variation in their musculature, mechanoreceptor numbers and bristle morphology. Overall, species with short, thin, branching bristles that lacked mechanoreceptors tended to forage pre‐dusk in open habitats, whereas species with mechanoreceptors around their bristle follicle tended to forage at night and in more closed habitats. We suggest that rictal bristles are likely to be tactile in many species and may aid in navigation, foraging and collision avoidance; however, identifying rictal bristle function is challenging and demands further investigation in many species.

## Graphical abstract

Rictal bristle morphology and follicle anatomy are diverse across certain Caprimulgiform species. Species with short, thin, branching bristles that lack mechanoreceptors tend to forage pre‐dusk in open habitats, whereas species with longer rictal bristles and mechanoreceptors around their bristle follicle tend to forage at night and in more closed habitats.

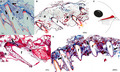

## 1 INTRODUCTION

Across the animal kingdom, the sense of touch is one of the most specialised senses (Kidd, 1907), yet it remains one of the least studied and understood (Prescott and Dürr, 2015). This is especially true in birds, despite birds having touch‐sensitive beaks (Gerritsen and Meiboom, 1985; Schneider *et al.*, 2014), and their feathers also being sensitive to vibrations and touch (Stettenheim, 1972; Seneviratne and Jones, 2008). Unlike mammalian hair, bird feathers have complex morphologies associated with function (Chuong *et al.*, 2000); including the well‐developed plumulaceous and pennaceous contour feathers, and filoplumes, which have a long rachis and a tuft of barbs at the tip (Stettenheim, 2000). Avian rictal bristles have the simplest external structure of all feathers (Stettenheim, 1973). They are modified hair‐like feathers composed of a single rachis, which sometimes also has venation at the base (Stettenheim, 1972). Feathers consist of ‘dead cells‘ that are enclosed at their base by an innervated follicle, thus resembling mammalian whiskers (Lederer, 1972; Pass, 1989). While the function of mammalian whiskers is well described—they are prominent tactile sensors that are used to guide locomotion, navigation, foraging and social interactions (Mitchinson *et al.*, 2011; Grant and Arkley, 2016)—the function of rictal bristles remains unclear (Cunningham *et al.*, 2011).

Bristles may serve to increase the size of a bird’s effective mouth gape (Van Tyne and Berger, 1976) or protect their eyes from airborne particles, prey items (Dyer, 1976; Conover and Miller, 1980) or vegetation (Brush, 1967; Hill, 1968). Rictal bristles may also prevent soiling of facial feathers during feeding (Chandler, 1914) or serve as a tactile organ to aid in navigation and obstacle avoidance (Küster, 1905; Lucas and Stettenheim, 1972). The position of the bristles above and along the upper beak, in front of the eye, may enable the bristles to ‘bounce’ flying insect legs or wings away from the eyes and into the mouth (Jackson, 2007). Experiments on Willow Flycatchers (*Empidonax traillii*) have revealed that rictal bristles can protect the eyes from prey particles, as taping around the bristles increased the number of small particles colliding with the eye (Conover and Miller, 1980). Experimental studies have also revealed that the rictal bristles of several other insectivorous neotropical tyrant‐flycatchers (*Myiarchus crinitus*,* Sayornis phoebe*,* Contopus virens*,* Sarcophaga bullata*, *Empidonax traillii*) did not appear to play a role in prey capture, as these species captured prey items between the tip of the mandibles without the prey making contact with the bristles (Lederer, 1972; Conover and Miller, 1980). Conversely, it has been suggested that Brown Kiwis (*Apteryx mantelli*) use their bristles to guide nocturnal foraging for subterranean prey, due to their whiskers being sensitive to vibrations (Cunningham *et al.*, 2007, 2013). The prevalence of bristles in nocturnal and crepuscular bird species suggests that rictal bristles may play a role in navigation and obstacle avoidance in low‐light conditions (Lucas and Stettenheim, 1972), but evidence for this is scant.

Anatomical studies conducted on the rictal bristles of owls (*Bubo bubo*,* Asio flammeus*,* Athene noctua* and *Strix aluco*) have revealed that their follicles are connected by muscle and connective tissue within the dermis, and are associated with nerves and mechanoreceptors (Küster, 1905; Stettenheim, 1973). Similarly, the follicles of the rictal bristles of Brown Kiwis are surrounded by Herbst corpuscles (Cunningham *et al.*, 2013), which are vibration‐sensitive mechanoreceptors. The presence of Herbst corpuscles indicates that rictal bristles are likely to be sensitive to touch, airflow and vibrations. Feather follicles are connected to each other by several muscles, notably by the smooth apterial muscle (counteracting horizontal movements of feathers), the smooth erector muscle (which lifts the feather up) and the depressor muscle (which pulls the feather down and counteracts the vertical rotation of feathers induced by airflows) (Ostmann *et al.*, 1963). Bristle follicles may also be connected to each other and have the capacity to be mobile; however, bristle anatomy has been described in only a handful of bird species, and we know very little about rictal bristle mechanoreceptors and musculature. Further histological work is therefore required to describe rictal bristle anatomy and to test hypotheses about their function. In mammals, longer and more numerous whiskers are found in small, nocturnal, arboreal species. These species have regularly arranged whisker follicles with large, regular intrinsic muscles (Muchlinski *et al.*, 2013; 2020; Grant *et al.*, 2017); longer, thicker and stiffer whiskers also tend to have more numerous mechanoreceptors (Ebara *et al.*, 2002). Whereas the anatomy of mammalian whiskers is well described, the morphology and anatomy of avian rictal bristles are relatively unknown, and the relationship between bristle morphology and anatomy has not previously been investigated.

In this study, we focus on bird species belonging to the Order Caprimulgiformes, commonly referred to as nightjars, which are widely known for their nocturnal ecology, with species exhibiting a highly diverse range of foraging traits. Many Caprimulgiform species have obvious and prominent rictal bristles that vary in their shape, length, number and thickness. Here we describe the bristle morphology and follicle anatomy of 12 species, representing all five families and nine of the 22 genera of the traditional Caprimulgiformes (Cleere, 1998). We go on to describe follicle anatomy by identifying the presence and prevalence of mechanoreceptor Herbst corpuscles and provide muscle fibre descriptions. We explore associations with bristle morphology traits (bristle length, width and number) and discuss our findings in light of recent data on phylogenetic relationships and known foraging traits.

## 2 MATERIALS AND METHODS

### 2.1 Samples

Rictal bristle morphology and follicle anatomy were described in 12 species belonging to the Caprimulgiformes order in this study: fiery‐necked nightjar (*Caprimulgus pectoralis*), pennant‐winged nightjar (*Caprimulgus vexillarius*), European nightjar (*Caprimulgus europaeus*), spotted nightjar (*Eurostopodus argus*), Australian owlet‐nightjar (*Aegotheles cristatus*), nacunda nighthawk (*Chordeiles nacunda*), common nighthawk (*Chordeiles minor*), tawny frogmouth (*Podargus strigoides*), large frogmouth (*Batrachostomus auritus*), Gould’s frogmouth (*Batrachostomus stellatus*), oilbird (*Steatornis caripensis*), pauraque (*Nyctidromus albicolis*). *Caprimulgus pectoralis* and *Caprimulgus vexillarius* specimens were donated by Professor Tim Birkhead at the University of Sheffield; they were decapitated and preserved in formalin prior to their donation. All the other specimens were from the spirit collection at the Natural History Museum, Tring, UK, where they were dissected and preserved in 4% paraformaldehyde (PFA) in phosphate buffer solution (PBS). The rictal bristle region was fully intact in all specimens and thus representative of the morphological characteristics of each species, with the exception of *B. auritus*, whose bristles were damaged prior to collection. All work in this study was approved by the local ethics committee at Manchester Metropolitan University.

### 2.2 Bristle morphology

**Figure 1:**
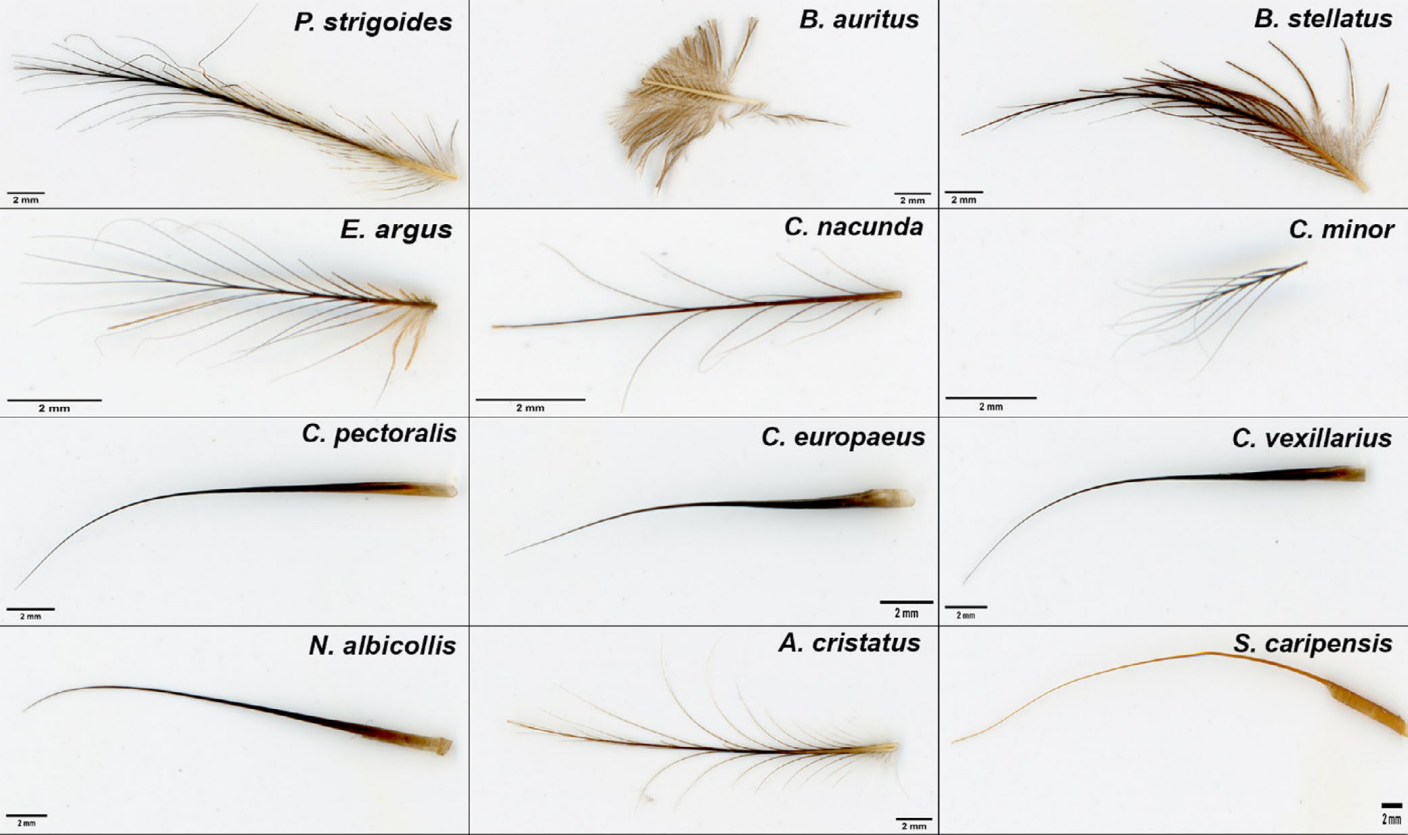
Scan of 12 rictal bristles showing the different bristle morphologies present in the Caprimulgiform order. Rictal bristles illustrated are from the longest rictal bristles found for each of our 12 species. Scale bar: 2 mm

For each species, the three longest rictal bristles were scanned on an Epson V600 scanner (12,800 dpi). Photos were taken using the Epson scan v 3.9.2 software, which gave calibrated measurements of the bristles. We were unable to photograph the bristles of *B. auritus* due to some of the bristles being damaged, and consequently we scanned only the base of rictal bristles for this species (see Figure 1). Bristle total length and bristle width at the base and tip were measured using the segmented line tool on imagej software from the scanned images. To compare the different morphotypes of bristles across species, we used three categories of bristle length: short (< 10 mm), medium (15–30 mm) and long (> 30 mm). Bristle width was also classified into three categories: thin (< .3 mm), moderately thick (.3–.6 mm) and thick (> .6 mm). Bristle number was counted from the rictal region of two individual specimens per species from the skin collection within the Natural History Museum, Tring, and Liverpool World Museum. As the number of bristles did not vary between each side of the head of each specimen, we only conducted bristle counts on one side of the face. Bristle numbers were categorised into few (≤ 10) and many (> 10).

### 2.3 Follicle anatomy

#### 2.3.1 Dissection and histology

**Figure 2:**
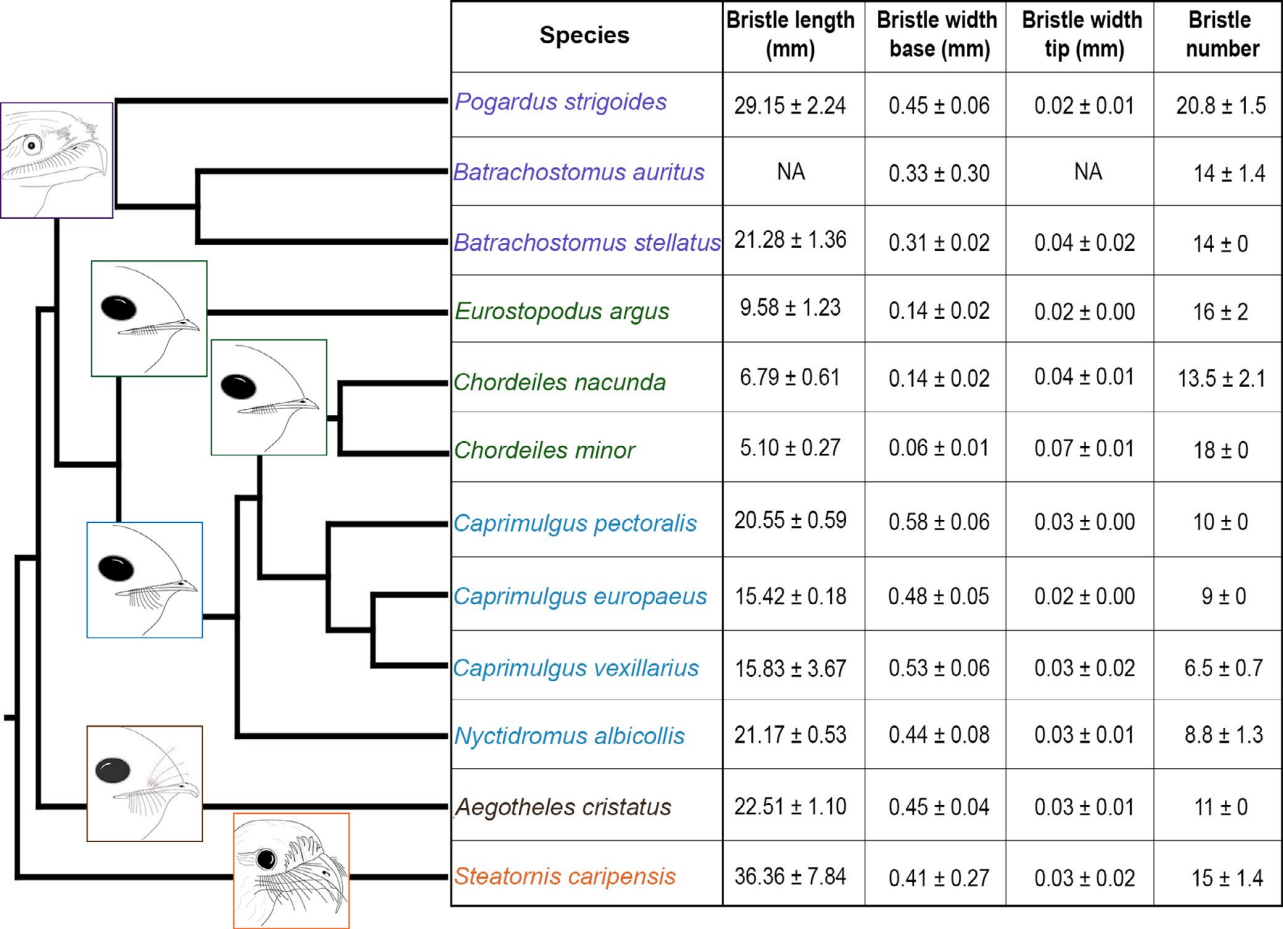
Phylogenetic tree for the species belonging to the Caprimulgiformes order used in this study, combined with a table showing their bristle measurements. Drawings showing the bristle morphology and position on the beak were added on the tree for better visualisation, and also to illustrate the head and beak shapes of the species. The tree was created by BirdTree.org. Measurements were taken from scanned bristles from each species and included the mean bristle length, mean bristle width at the base and at the tip (± standard deviation). Species names are colour‐coded following their bristle morphotypes: frogmouths in purple, nighthawks and spotted nightjar in green, nightjars and pauraque in blue, Australian owlet‐nightjar in brown, and oilbird in orange

We define the rictal region as the patch of skin from the rictus to nares, at the base of the upper mandible of the beak (Figure 2). Subsequently, we defined all bristles collected from this area as rictal bristles. All other (non‐bristle) feathers were trimmed from the rictal region of each specimen. The rictal bristle region was removed from one side of the head by cutting a 5‐mm‐wide piece of skin along the length of the upper beak and across the head near the nare using a scalpel. There was noticeable variation in bristle positioning across all specimens examined and consequently the shape and position of the dissected sections varied (Figures 4B, 5D, 6C and 7C,D). Rictal bristles were removed from the skin of each sample by cutting them flat to the skin sample; the base of the bristle was kept within the follicle to allow the follicle to maintain its form during slicing and staining.

Skin tissue was flattened for 5 hr between two sponges in histology cassettes in 70% industrial methylated spirit (IMS). Tissue samples were loaded into a tissue processor (Shandon Citadel 2000) to dehydrate through a graded series of ethanol (70, 80, 90 and 100%) and xylene baths and infiltrated with paraffin wax, in a process lasting approximately 20 hr. Tissue was then embedded in solid blocks of paraffin that were sliced at 10 µm on a rotary microtome (Thermo Scientific Microm HM355S) with water bath (37°C), and mounted onto slides, which were stained with standard Masson’s Trichrome staining and cover‐slipped with DPX mountant. Microscope images were taken using Zeiss zen pro imaging software on a Zeiss AxioImager M1 Brightfield microscope and AxioCam HMRc.

#### 2.3.2 Characterisation of follicle anatomy

For each species, components of the follicle and immediate surroundings of the follicle were identified and characterised from the microscope images, including describing the tissue type, musculature and mechanoreceptors. For each species, the dermis density and the adipose tissue quantity were quantified as possible predictors of tissue quality and functionality, using the following coding system. The quantity of adipose tissue was expressed as the percentage of space allocated for adipose tissue within the integument section imaged. Dense tissue was here defined as having ≤ 40% adipose tissue, and *porous* tissue was defined as having > 40% of adipose tissue within the histology section. Dense tissue may correspond to a higher quality of tissue, containing more sensory components. For each species, the presence and type of intrinsic fibres and mechanoreceptors were identified from the dermis. Types of intrinsic fibres were determined following the feather muscle description by Homberger and De Silva (2000) and defined as smooth apterial muscle, and erector or depressor feather muscle (the histology sections showed the characteristic diagonal fibres of the erector and depressor muscles, running from the tip of a feather follicle to the base of the neighbouring one, but the angle of the sections did not permit us to identify which of the depressor or erector muscles was present on the picture). The size of the fibre bundles was categorised by counting the number of fibres attached together in the area immediately surrounding the follicle; with ≥5 adjacent fibres considered a ‘large fibre bundle’, < 5 considered a ‘small fibre bundle’, and NA if no fibres were present. The presence/absence of mechanoreceptors was documented and, where present, the number of mechanoreceptors was counted per follicle and position. Based on our findings, we defined the number of Herbst corpuscles as low when their number around a follicle was < 7 and high when ≥ 7. Mechanoreceptor position was described as ‘base’ when they were only apparent at the proximal tip (base) of the bristle follicle, or ‘around’ when they were recorded from the base and the sides of the follicle. Mechanoreceptors were identified as Herbst corpuscle following Gottschaldt (1985). It was not possible to measure the depth of the bristle follicle due to variation in the orientation of the follicles within the tissue sample and the resulting histology slices.

#### 2.3.3 Relationship between bristle morphology, anatomy and foraging traits

Phylogenetic relationships of all nightjar species based on rictal bristle morphotypes (cf. drawings on phylogenetic branches in Figure 2) were represented using a phylogenetic tree from Birdtree.org (http://birdtree.org, tree set: HackettStage2Full, sample size: 1,000). Two k‐means cluster analyses were conducted in matlab (MATLAB and Statistics Toolbox Release 2019a, The MathWorks, Inc.) to identify morphotypes from our bristle morphology and follicle anatomy descriptors. The bristle morphology variables included the discrete categories of bristle length, width, number and branching, and the follicle anatomy variables included the discrete categories of muscle bundle size, tissue density and Herbst corpuscle number. Both datasets were partitioned into five defined groups using k‐means distance measures. Relationships between the numbers of Herbst corpuscles, bristle length, width and number were visualised using the package *ggplot2* (Wickham, 2009) in R software, version 3.5.0 (R Core Team, 2016) and analysed using non‐parametric Spearman’s rank correlations (due to low sample sizes and skewed data). All values are presented as mean values ± SD with significance level assumed at *p* < .05. Five ecological traits from Cleere (1998) were identified for each sampled species (Table S1) to compare and discuss these ecological traits in association with bristle morphology and follicle anatomy: foraging time, diet, foraging method, foraging height and habitat density. The traits are defined in detail in Table S1.

## 3 RESULTS

### 3.1 Bristle morphology

All three frogmouth species (*P. strigoides*,* B. auritus* and *B. stellatus*) had rictal bristles of medium length with a thick bristle base and a high number of bristles (Figure 2), which were conspicuously branched in structure (Figure 1). *Podargus strigoides* and *B. auritus* bristles were branched throughout with barbs and barbules, especially at the base, whereas *B. stellatus* had simple branching bristles with numerous barbs throughout (Figure 1). *Caprimulgus pectoralis*, *C. vexillarius*,* C. europaeus* and *N. albicollis* had medium length bristles with unbranched rictal bristles that occurred in lower numbers (≤10 bristles per side of the face) than the other species in the study (Figures 1 and 2). In contrast, *C. nacunda*,* C. minor* and *E. argus* had the thinnest and shortest rictal bristles, occurring in high numbers, and were branched with barbs (Figures 1 and 2). In all species with branched rictal bristles, *C. nacunda* had the least number of barbs on the shaft, from base to tip (Figure 1). *Aegotheles cristatus* had similar bristle lengths and widths to *C. pectoralis*,* C. vexillarius, C. europaeus* and *N. albicollis* (all with medium length and width); however, its bristles were a combination of branched with barbs from the base to the tip of the bristles and unbranched bristles, and had relatively lower bristle numbers (Figures 1 and 2). Finally, *S. caripensis* had thick, long, unbranched rictal bristles that curved downwards, in contrast to the bristles on all the other species, which curved slightly upwards at the distal tip (Figures 1 and 2), and had a high number of rictal bristles (15 bristles ± 1.4 SD) (Figure 2). Thus in our 12 species, bristle morphology could be visually summarised by five different morphotypes (coloured purple, green, blue, brown and orange in Figure 2), as confirmed by a k‐means cluster analysis (Table S2). Combining data from all the species sampled, bristle length and width were not correlated (Spearman’s rank correlation: *rho* = .36, *p* = .27; Figure S1), nor was bristle length correlated with bristle number (Spearman’s rank correlation: *rho* = .05, *p* = .88). However, bristle number and bristle width were correlated (Spearman’s rank correlation: *rho*= −.67, *p* = .016; Figure S1), suggesting that species with more bristles present, such as *C. nacunda, C. minor* and *E. argus*, also had much thinner bristles.

### 3.2 Follicle anatomy

#### 3.2.1 Characterisation of follicle anatomy

**Figure 3:**
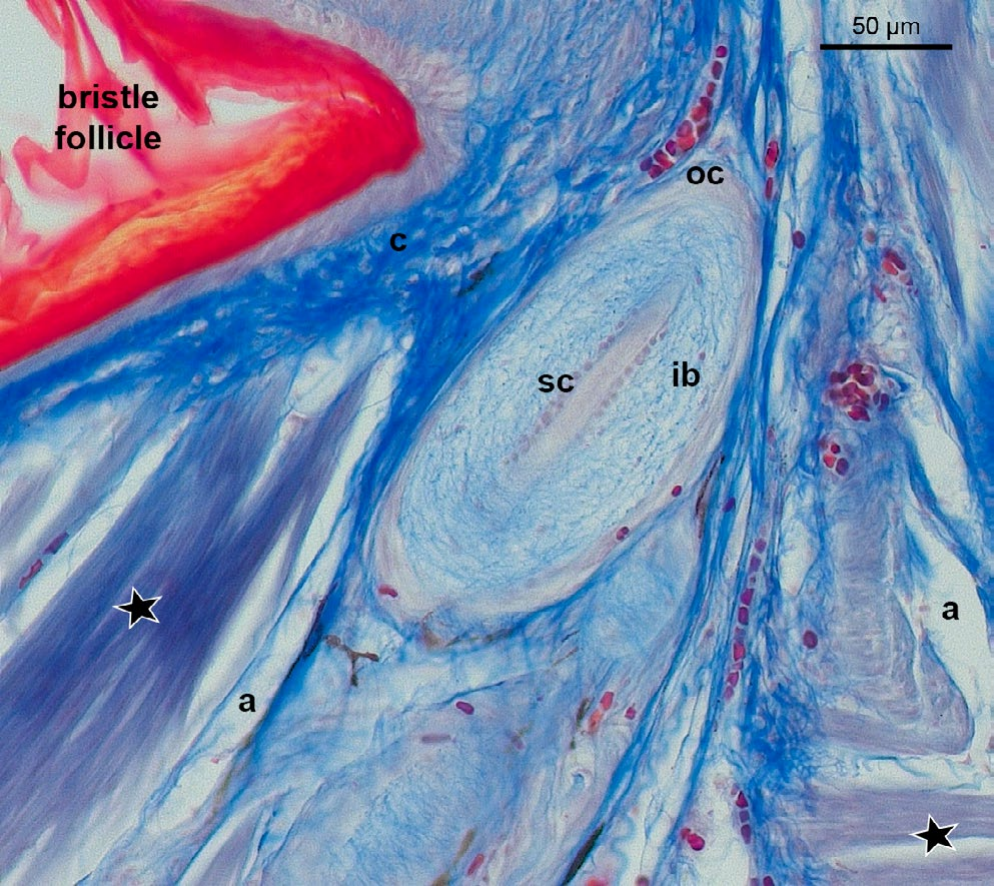
Longitudinal section of the dermis layer showing a cross‐section of Herbst corpuscles around a bristle follicle from the skin region above the upper beak of *C. pectoralis*. Star: muscle fibres. (a) adipose tissue; (c) connective tissue; (oc) outer capsule; (ib) inner bulb; (sc) rows of Schwann cells. Sections stained with Masson’s trichrome stain

Staining of the skin of the rictal region revealed high inter‐species variability of the bristle‐bearing integument, especially in the presence of Herbst corpuscle mechanoreceptors (Figure 3) and the intrinsic fibres in the surrounding of the bristle follicles (Table 1). There was no clear pattern in follicle anatomy morphotypes, based on a k‐means cluster analysis (Table S2).

**Table 1:** Summary table of the anatomical descriptions of the bristle‐bearing integument of the 12 species of the Caprimulgiform order

Species	Adjacent muscle	Fibre bundle	Connective tissue	HC position	HC number	Dermis density	Quantity of adipose tissue, %	Bristle number
*P. strigoides*	Smooth erector/depressor muscle smooth apterial muscle	Large	Yes	Around	4	Dense	20	20.8 ± 1.5
*B. auritus*	Smooth erector/depressor muscle smooth apterial muscle	Large	Yes	Around	5	Dense	10	14 ± 1.4
*B. stellatus*	Smooth erector/depressor muscle	Large	Yes	Around	5	Dense	30	14 ± 0
*C. nacunda*	Absent	Absent	Yes	Absent	0	Porous	70	13.5 ± 2.1
*C. minor*	Smooth erector/depressor muscle	Small	Yes	Absent	0	Porous	70	18 ± 0
*E. argus*	Smooth erector/depressor muscle	Small	Yes	Around	10	Porous	60	16 ± 2
*C. pectoralis*	Smooth apterial muscle	Large	Yes	Around	10	Dense	40	10 ± 0
*C. vexillarius*	Smooth erector/depressor muscle	Small	Yes	Absent	0	Porous	80	6.5 ± 0.7
*C. europaeus*	Smooth erector/depressor muscle	Small	Yes	Base	6	Porous	60	9 ± 0
*N. albicollis*	Smooth erector/depressor muscle	Large	Yes	Around	5	Dense	20	8.8 ± 1.3
*A. cristatus*	Smooth erector/depressor muscle	Large	Yes	Around	8	Dense	40	11 ± 0
*S. caripensis*	Striated subcutaneous muscle	Large	Yes	Around	10	Dense	10	15 ± 1.4

Note

HC position corresponds to the position of Herbst corpuscles at the bristle follicle. Bristle number corresponds to the number of bristles on one side of the face. Species names colour‐coded following their bristle morphotypes, based on bristle size (length and width), shape (branching present and absent) and number (not follicle anatomy): frogmouths in purple, nighthawks and spotted nightjar in green, nightjars and pauraque in blue, Australian owlet‐nightjar in brown, and oilbird in orange.

HC, Herbst corpuscles.

**Figure 4:**
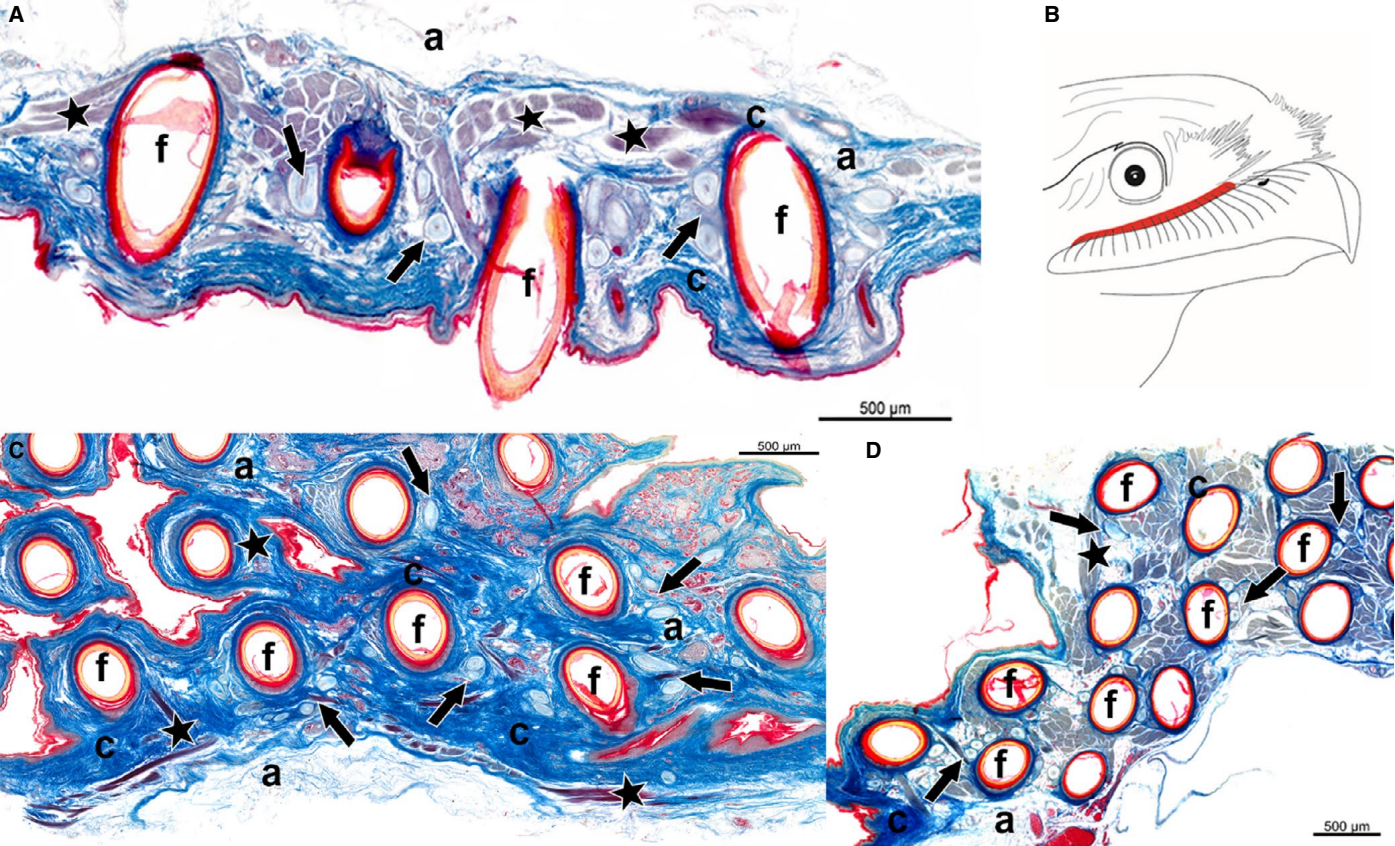
Longitudinal section of the dermis layer showing a cross‐section of bristle follicles from the skin region above the upper beak of (A) *P. strigoides*, (C) *B. auritus* and (D) *B. stellatus*, from the nares to the rictus (right to left). Sections revealed the presence of mechanoreceptors, i.e. Herbst corpuscles (arrows), muscle fibres (stars), and adipose tissue (a) surrounding the bristle follicles (f). Sections stained with Masson’s trichrome stain. (B) Schematic drawing illustrating in red the correspondent skin region cut along the upper beak, enclosing the bristle follicles of *P. strigoides, B. auritus* and *B. stellatus*

The bristle‐bearing integument of *P. strigoides, B. auritus* and *B. stellatus* revealed a dense tissue (Figure 4, Table 1) with the smooth erector or depressor muscles running diagonally from consecutive bristle follicles. In *P. strigoides* and *B. auritus*, the smooth apterial bristle muscle was also present, joining the distal tips of bristles together (Figure 4). In all three specimens, the dermis contained connective tissue around the rictal bristle follicles (shown as a blue ring around the follicle; Figure 4). Fewer Herbst corpuscles mechanoreceptors were present in the surroundings of the rictal bristle follicles (Figure 4, Table 1). Herbst corpuscle structure possessed a characteristic outer capsule enclosing an onion‐shaped lamellate outer zone and an inner bulb, surrounded by two rows of Schwann cell nuclei (Figure 3).

**Figure 5:**
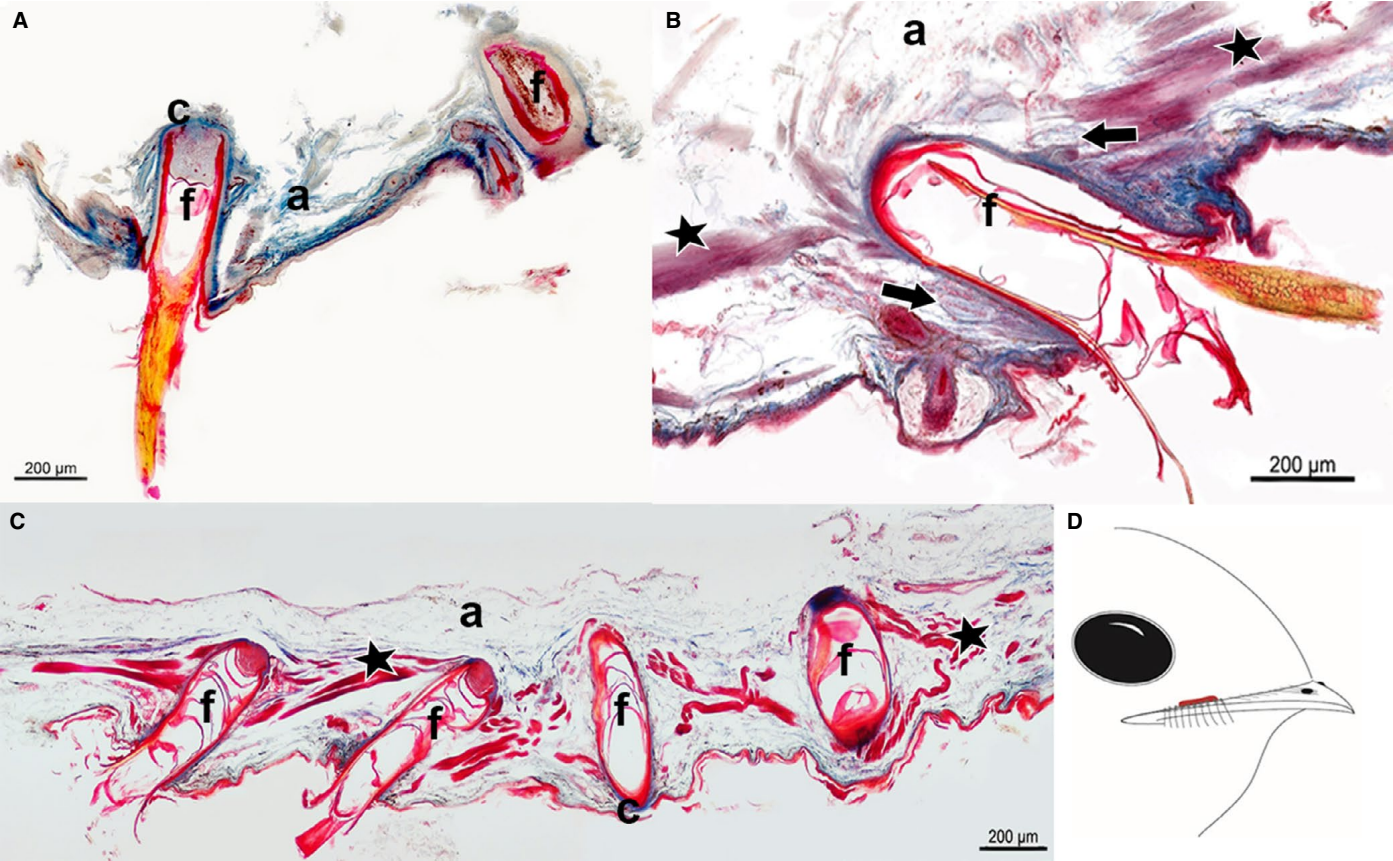
Longitudinal section of the dermis layer showing a cross section of bristle follicles from the skin region above the upper beak of (A**)** *C. nacunda,* (B**)** *E. argus* and (C**)** *C. minor*, from the nares to the rictus (right to left). Sections revealed the presence muscle fibres (stars), adipose tissue (a), and mechanoreceptors, i.e. Herbst corpuscles (arrows) only in *E. argus*, surrounding the bristle follicles (f). Sections stained with Masson’s trichrome stain. (D) Schematic drawing illustrating in red the correspondent skin region cut along the upper beak, enclosing the bristle follicles of *C. nacunda, C. minor* and *E. argus*

The integument of *C. nacunda*, *C. minor* and *E. argus* revealed porous tissue within the dermis (Figure 5, Table 1). Samples from all three species had bristle follicles enclosed in connective tissue (Figure 5). *Chordeiles nacunda* had no intrinsic fibres present in the surroundings of the bristle follicles, but *C. minor* and *E. argus* had small bundles of the smooth erector or depressor muscle fibres extending from the base of a follicle to the neck of a neighbouring one (Figure 5, Table 1). There were no mechanoreceptors present in the tissue immediately surrounding the rictal bristles in *C. nacunda* or *C. minor*, whereas a high number of Herbst corpuscles were found in the tissue surrounding the rictal bristle follicles in *E. argus* (Figure 5, Table 1).

**Figure 6:**
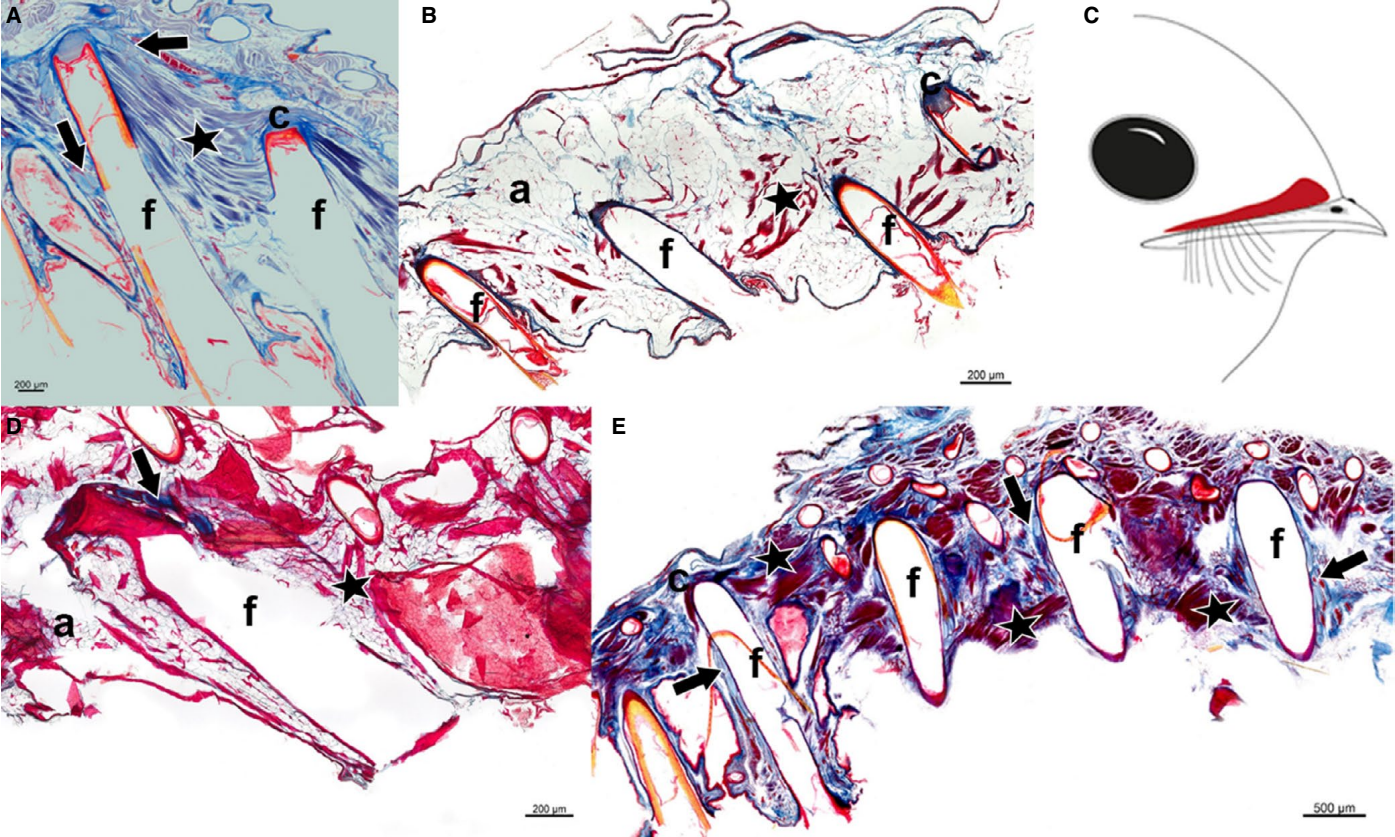
Longitudinal section of the dermis layer showing a cross section of bristle follicles from the skin region above the upper beak of (A) *Caprimulgus pectoralis,* (B) *Caprimulgus vexillarius,* (D) *Caprimulgus europaeus* and (E) *Nyctidromus albicollis*, from the nares to the rictus (right to left). Sections revealed the presence of mechanoreceptors, i.e. Herbst corpuscles (arrows), muscle fibres (stars), and adipose tissue (a) surrounding the bristle follicles (f). Sections stained with Masson’s trichrome stain. (C) Schematic drawing illustrating in red the correspondent skin region cut along the upper beak, enclosing the bristle follicles of *P. strigoides, B. auritus* and *B. stellatus*

There was interspecific variation in the musculature, mechanoreceptors and density of the bristle‐bearing integument in *C. pectoralis, C. vexillarius, C. europaeus* and *N.* *albicollis* (Figure 6). *Caprimulgus pectoralis* displayed a dense tissue (Figure 6A, Table 1), with a dermis presenting rictal bristle follicles connected together by a dense layer of smooth apterial muscle (Figure 6A). A high number of mechanoreceptors were present all around the rictal bristle follicles in *C. pectoralis* from the distal tip of the follicle (Figure 6A, Table 1). The skin tissue of *C. vexillarius* revealed a porous integument, in which small bundles of smooth erector or depressor muscles fibres connected the bristle follicles together (Figure 6B; Table 1). There was no evidence of mechanoreceptors in the bristle‐bearing integument of *C. vexillarius* (Figure 6B, Table 1). The integument of *C. europaeus* consisted of a porous dermis with a small bundle of smooth erector or depressor muscle fibres that appeared to connect bristle follicles together, and a thin layer of connective tissue was apparent at the base of the bristle follicles (Figure 6D; Table 1). In addition, a small number of Herbst corpuscles were present around the rictal bristle follicles, constricted at the base of the follicle (Figure 6D, Table 1). The integument of *N. albicollis* revealed dense tissue and a large bundle of smooth erector or depressor muscle fibres, pairing bristle follicles together (Figure 6E, Table 1). The follicles were also surrounded by connective tissue, in which the muscle fibres seemed to be attached. Herbst corpuscles were present all around the follicles, from the distal tip to the neck of the follicle (Figure 6E); however, the number of mechanoreceptors in *N. albicollis* was low compared with the other species (Figure 6E, Table 1).

**Figure 7:**
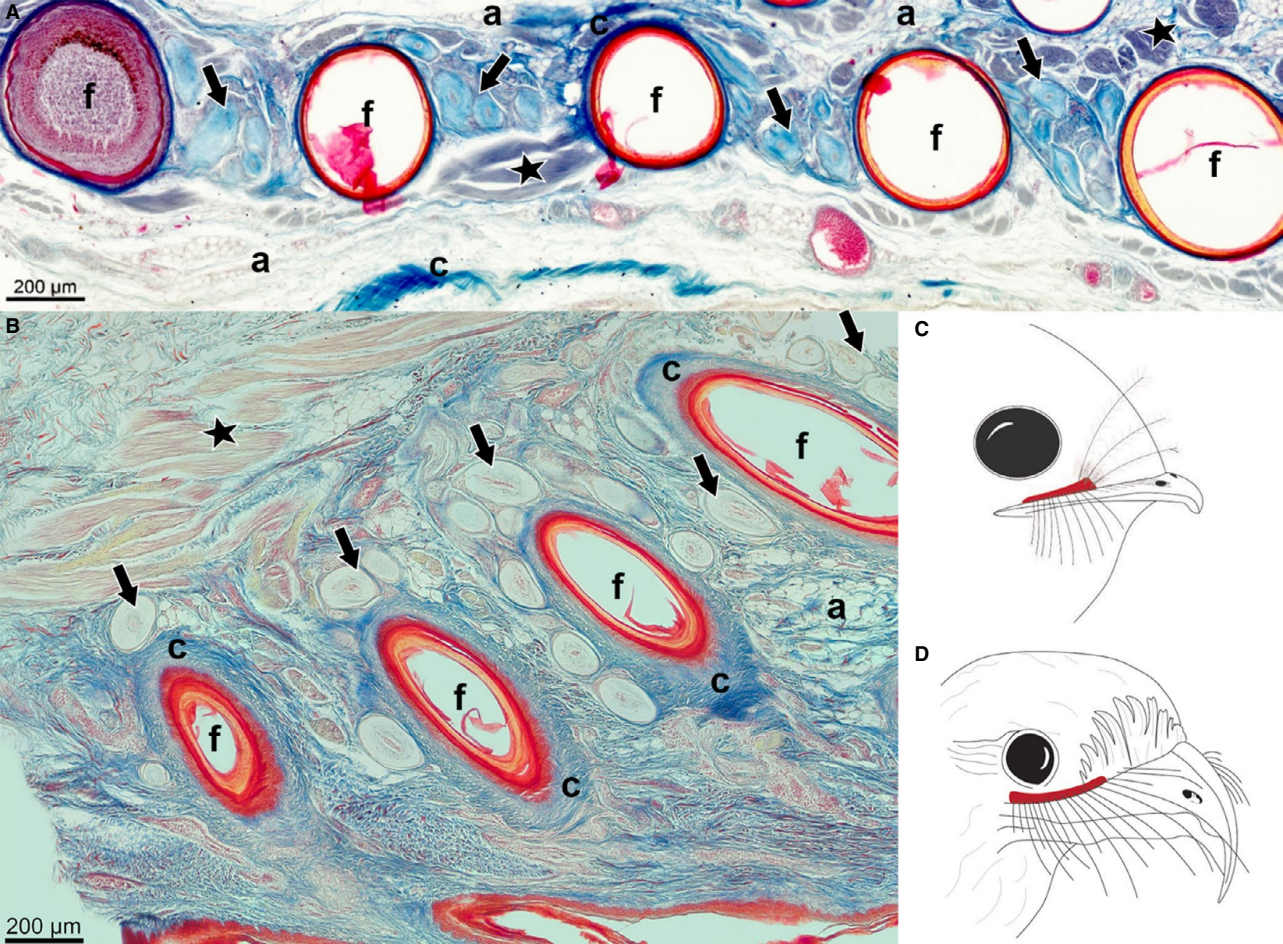
Cross‐ (A) and longitudinal section (B) of bristle and feather follicles from the dermis layer of the rictal region above the upper beak of (A) *Aegotheles cristatus* and (B) *Steatornis caripensis*, from nare to rictus (right to left). Sections revealed the presence of mechanoreceptors, i.e. Herbst corpuscles (arrows), muscle fibres (stars), and adipose tissue (a) surrounding the bristle follicles (f). Sections stained with Masson’s trichrome stain. Schematic drawing illustrating in red the skin region cut along the upper beak, enclosing the bristle follicles of (C) *A. cristatus* and (D) *S. caripensis*

The sections from the *A. cristatus* specimen had a different orientation (angle) to the other samples and did not show the same longitudinal section of the bristle follicle. *Aegotheles cristatus* had dense tissue (Figure 7A, Table 1), with large bundles of the smooth erector or depressor muscle fibres, pairing the bristle follicles together. Furthermore, the bristle follicle appeared to be surrounded by connective tissue and Herbst corpuscles (Figure 7A). Despite the different orientation (angle), a high number of Herbst corpuscles around the follicles were distinguishable (Figure 7A, Table 1). The integument of *S. caripensis* consisted of a dense tissue, with a large sheet of striated subcutaneous muscle fibres underlying the bristle follicles (Figure 7B, Table 1). *Steatornis caripensis* had abundant connective tissue around the follicle, as well as numerous Herbst corpuscles (Figure 7B, Table 1).

#### 3.2.2 Relationship between bristle morphology and follicle anatomy

Herbst corpuscle number was not significantly correlated with bristle length (Spearman’s Rank: *rho* = .39, *p* = .23) or bristle width (Spearman’s rank correlation: *rho* = .23, *p* = .47) (Figure S1). However, *S. caripensis,* which had the longest bristles, also had the highest number of mechanoreceptors, while in the two *Chordeiles* species, which had the shortest and thinnest bristles, had no mechanoreceptors present (Table 1). While we were not able to test this statistically, small bundles of muscle fibres tended to be present in species with short (*C. minor* and *E. argus*) and medium‐sized bristles (*C. vexillarius* and *C. europaeus*), whereas large muscle bundles were present in species with medium or long bristles (Table 1). Herbst corpuscle number was not correlated to bristle number (Spearman’s rank correlation: *rho*= −.03, *p* = .93; Figure S1). Therefore, although there are some anecdotal associations of bristle morphology and follicle anatomy in our data, quantitative measurements of bristle length, width and number were not correlated with the number of Herbst corpuscles.

## 4 DISCUSSION

### 4.1 Bristle morphology

We identified five different bristle morphotypes in the 12 Caprimulgiformes species investigated in this study (Figure 2, Table S2), which varied in bristle size (length and width), shape (branching present and absent) and number. These morphotypes seemed to be in accordance with a contemporary Caprimulgid phylogeny (Figure 2, Table S2), where closely related species had similar bristle morphologies. We also noticed that the beak and head shapes of the species affected the position of the bristles, and hence the dissection area (Figures 4, 5, 6, 7). Beak shape is one of the most diverse traits in birds, varying in morphology according to individual species and their feeding strategies (Stettenheim, 2000; Cunningham *et al.*, 2013; Thomas *et al.*, 2016). Due to the small sample sizes and specimen availability of the species in this study, we are not able to conduct phylogenetic analyses; however, we demonstrate below that bristle morphology does not coincide consistently with beak shape and foraging traits, rather it is distributed non‐randomly over the contemporary Caprimulgid phylogeny.

Seven of our sampled species (*C. pectoralis, C. vexillarius, C. europaeus, N.* *albicollis*, *C. nacunda, C. minor* and *E. argus*) are predominantly insectivorous, with small, thin beaks and a wide mouth gape (Cleere, 1998). However, although the aforementioned species share a similar beak shape and diet, they vary widely in their bristle morphology, including length, width and branching. Hence, *C. pectoralis, C. vexillarius, C. europaeus *and* N. albicollis* appear to be in a group distinct from *C. nacunda, C. minor* and *E. argus*, in terms of both bristle morphology and phylogenetically. Although *A. cristatus* has the same diet as the seven previous species, it has a slightly more keratinised and hooked beak than the others (Cleere, 1998) and possesses a combination of unbranched and branched bristles. There are also examples in our specimens where a congruence seems to appear between bristle morphology, beak shape, foraging traits and phylogeny. *Podargus strigoides, B. auritus* and *B. stellatus* all shared similar beak shape, diet (Cleere, 1998) and bristle morphology and thus form a sister group, as these features differed from our other species (Figure 2). *Steatornis caripensis* has a different beak shape, diet (Cleere, 1998) and bristle morphology than all the other species (Figure 2), consequently *S. caripensis* is rather distinct from the others and is on its own within the phylogeny. Measuring the bristle morphology of more specimens and species will allow us to explore these relationships further, especially using phylogenetic analyses.

### 4.2 Follicle anatomy

For all species examined in this study, we found that bristle follicles were anchored in the dermis of the skin with an outermost layer of connective tissue, which is consistent with previous studies on avian rictal bristles (Ostmann *et al.*, 1963; Homberger and De Silva, 2000). The appearance of muscle fibres connecting the follicles has also been observed in contour feathers, such as in the wild turkey (*Meleagris gallopavo*) (Homberger and De Silva, 2000; 2003). The size and arrangement of smooth muscles at the follicles have been found to vary between feather types, i.e. large flight feathers have larger muscle bundles then small filoplumes, which have no muscles (Stettenheim, 2000). In agreement with this latter study, we also found that short, thin bristles had smaller muscle bundles than medium or long, wide bristles (Figure 2, Table 1).

Bristle morphology was not associated with follicle anatomy in the species in this study (Figures 1, 2, 3, 4, 5, 6, 7), as there was no correlation between the presence of muscles and the number of Herbst corpuscles, and bristle length, width or number. In mammalian whiskers, whisker length, width and number are associated with aspects of follicle anatomy and musculature (Muchlinski, 2010; Muchlinski *et al.*, 2013; Grant *et al.*, 2017); specifically, longer and more numerous whiskers tend to be associated with more arranged follicles and large intrinsic muscles (Muchlinski, 2010; Muchlinski *et al.*, 2013; Grant *et al.*, 2017), and longer, thicker and stiffer whiskers tend to have numerous mechanoreceptors (Ebara *et al.*, 2002). However, this was not the case in our species. For example, *C. pectoralis, C. vexillarius* and *C. europaeus* had differences in follicle anatomy from one another, in terms of the size of muscle fibre bundles and mechanoreceptors, although they had the same bristle morphology. While bristle morphology visually agreed with the contemporary Caprimulgiform phylogeny (Figures 1 and 2), the follicle anatomy (musculature and Herbst corpuscle number) did not. If this is the case, bristle follicle anatomy, and hence tactile sensitivity, may be better explained by life‐history traits, rather than phylogenetic relatedness. This has not been explored across a comparative dataset before and should form the basis of future studies on bristle anatomy and morphology.

### 4.3 Foraging traits and bristle function

The Caprimulgiform order is ecologically and behaviourally diverse, with species exhibiting different activity patterns, diet, foraging methods, foraging niches and patterns of habitat selection (Table S1) (Cleere, 1998). We suggest below that foraging time, foraging habitat selection and foraging method might all be associated with follicle anatomy in the Caprimulgiformes species that we studied here; specifically, tactile sensitive rictal bristles (with mechanoreceptors) are present in the Caprimulgiform species that forage in scotopic conditions in more closed, structurally complex habitats, whereas bristles without mechanoreceptors are present in our Caprimulgiformes species that are open habitat, partially diurnal foragers.

Three of our study species that forage pre‐dusk (*C. nacunda, C. minor* and *C. vexillarius*) had no mechanoreceptors present around their rictal bristle follicles, and had only short to medium length bristles (Figures 5, 6,C and 6B Table 1). The anatomy morphotype k‐means cluster analysis also grouped these species together (Table S2). Although *C. vexillarius* is found in semi‐open habitats, whereas *C. nacunda* and *C. minor* are found in open habitats, all three species forage by hawking high above the canopy (in the case of *C. vexillarius*) or in open country or riverbeds (in the case of *C. nacunda* and *C. minor*) (Tables 1 and S1; Holyoak, 2001). Therefore, open‐habitat, partially diurnal foraging species seem to have reduced bristle tactile function. The lack of mechanoreceptors suggests that these species may rely more on vision than touch, as flying during daylight in open habitats probably makes them less likely to collide with obstacles and more likely to detect prey visually. Rictal bristles in these species may still play a role in eye protection against flying items during feeding, despite them being less than 20 mm in length (Lederer, 1972; Conover and Miller, 1980). In addition, the bristles may also still be sensitive, as their movement could be detected in the absence of Herbst corpuscles by other mechanoreceptors, e.g. stretch receptors such as Ruffini corpuscles, which detect pressure and tension and are present in the muscle and skin (Halata and Munger, 1980).

Contrary to these partially diurnal species, we found that all nine obligate crepuscular and nocturnal species had mechanoreceptors present around their bristle follicles and so are likely to have a tactile function. These species all forage in complex or closed habitats, which suggests that bristles may be involved in foraging, navigation and collision avoidance in the dark. The evolution of avian foraging traits often depends upon a trade‐off between vision and other sensory systems (Cunningham *et al.*, 2013; Corfield *et al.*, 2015), therefore, touch sensing might well be more developed in nocturnally foraging species, as is the case in mammals (Muchlinski, 2010; Muchlinski *et al.*, 2013). The number of mechanoreceptors located around the follicle varied across our species, and we observed no clear relationship between the number of Herbst corpuscles and the anatomy morphotypes, with their foraging traits—the status of obligate crepuscular and nocturnal, and the density of their habitat (Table S1). This indicates that the sensitivity of rictal bristles is likely to be dependent upon a combination of species‐specific life‐history traits. For example, two obligate nocturnal species, *B. auritus* and *B. stellatus*, which prefer close habitats such as densely vegetated forest habitats, had low numbers of mechanoreceptors, whereas near‐obligate nocturnal species *C. pectoralis, S. caripensis* and *E. argus*, had the highest number of Herbst corpuscles present, and live in both semi‐open and closed habitat. The near‐obligate nocturnal *C. europaeus*, which prefers semi‐open habitat (i.e. a mixture of open country and woodlands, Table S1), had low numbers of mechanoreceptors present at the base of the bristle follicle (Table S1). *Caprimulgus europaeus,* like the partially diurnal species that lacked mechanoreceptors, is also an aerial hawking species (Cleere, 1998). Therefore, sensitive, tactile rictal bristles may not be beneficial for species that predominantly feed by aerial hawking.

Feathers have previously been found to guide navigation and foraging in a number of species. For example, the crest and super‐orbital plumes in whiskered auklets (*Aethia pygmaea*) are used to detect obstacles and guide navigation in dark crevices (Seneviratne and Jones, 2008). Brown kiwi (*Apteryx mantelli*) are nocturnal birds that have long rictal bristles, with a mean of 36.63 mm (SD 7.97, *n* = 1 individual, 6 bristles) (M. G. Delaunay unpubl. data), which are longer than any of the bristles in our study species, and also possess numerous (>10) Herbst corpuscle mechanoreceptors positioned all around the follicles (Figure 3 in Cunningham *et al.*, 2011), which is similar to the number in *E. argus*, *C. pectoralis* and *S. caripensis* in our study (Figures 5B, 6A and 7B). Cunningham *et al., *(2011) suggest that, as kiwis probe‐forage holding their bristle forwards, they may use their tactually sensitive rictal bristles to detect subterranean prey or to assess the quality of the foraging ground. Therefore, rictal bristles of *E. argus, C. pectoralis* and *S. caripensis* may be functionally similar and help to detect flying prey or obstacles during flight. Corfield *et al.* (2014) also proposed that brown kiwi rictal bristles are tactile and might help with navigation in the dark.

Defining the function of rictal bristles is complex and their sensitivity is likely to be dependent upon a combination of species‐specific life‐history traits. This is the case in mammalian whiskers, where aquatic mammals have more sensitive whiskers with 10× more nerve endings than terrestrial mammals (Hyvärinen and Katajisto, 1984; Hyvärinen, 1989; Marshall *et al.*, 2006), whereas arboreal, nocturnal small mammals have larger and more regular whisker muscles (Grant and Arkley, 2016; Muchlinski *et al.*, 2020); although many aspects of muscle and whisker arrangements can still vary on a species‐by‐species basis (Yohro, 1977; Grant *et al.*, 2013, 2017). Further work investigating more species and specimens across Caprimulgiformes and other avian orders will help to develop our understanding of bristle function, specifically addressing which ecological variables might predict the presence of long, sensitive bristles. However, the morphology and anatomy of the rictal bristles are not closely associated. This means that morphological variables cannot infer tactile sensitivity, and conducting large‐scale morphological studies, using museum skin collections for instance, will have more limited applications. Rictal bristles are present in many avian species, not only in the Caprimulgiformes, and investigating the bristle morphology and follicle anatomy in more diverse species could help us better define the function and evolution of rictal bristles.

## AUTHOR’S CONTRIBUTIONS

R.G. and M.D. designed the study and drafted the manuscript. M.D. performed the histology, did the measurements and made the figures. C.L. helped getting access to the spirit collection of the Natural museum of Tring and the skin collection of the World museum of Liverpool. Analyses and interpretations were performed by M.D. and critically reviewed by R.G., C.L., H.L. and M.S. All authors revised the manuscript and provided final approval before submission.

## Supporting information

Fig S1

Supplementary Material

## Data Availability

We have supplied the data for each species (mean and SD); these are available in the tables of the manuscript.
