# Supplementary figures and images for: Anatomy of avian rictal bristles in Caprimulgiformes reveals reduced tactile function in open‐habitat, partially diurnal foraging species
Source: J Anat. 2020 Mar 23;237(2):355–66. doi: 10.1111/joa.13188 (PMC7369198; doi:10.1111/joa.13188)

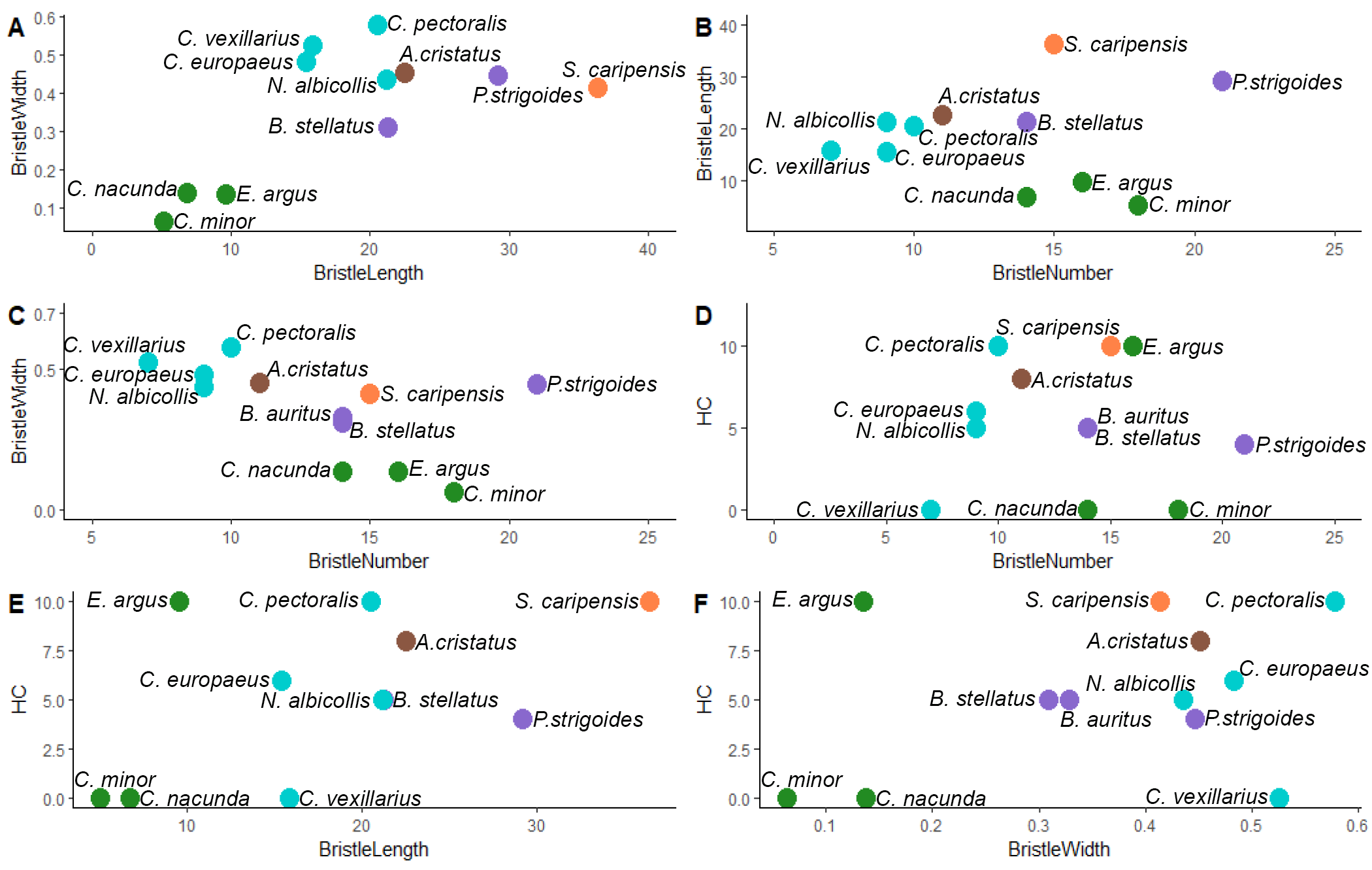

Supplement: Supplementary file 1 — Fig S1 [file JOA-237-355-s001.tif]
