# Supplementary material for: Anatomy of avian rictal bristles in Caprimulgiformes reveals reduced tactile function in open‐habitat, partially diurnal foraging species
Source: J Anat. 2020 Mar 23;237(2):355–66. doi: 10.1111/joa.13188 (PMC7369198; doi:10.1111/joa.13188)
Supplement: Supplementary file 2 — Supplementary Material [file JOA-237-355-s002.docx]

**Fig. S1. Scatterplots** **of A) Bristle length against Bristle width, B) Bristle number against Bristle length, C) Bristle number against Bristle width, D) Bristle number against Herbst corpuscle number, E) Bristle length against Herbst corpuscle number, and F) Bristle width against Herbst corpuscle number.** Species are coloured coded following their bristle morphology: frogmouths in purple, nighthawks and spotted nightjar in green, nightjars and pauraque in blue, Australian owlet-nightjar in brown and oilbird in orange.

## Table S1. Ecological factors of interest for each species of the study, based on Cleere (1998). Foraging time includes: partially diurnal (birds active before dusk), crepuscular (birds active at dusk and dawn), and nocturnal (during the night). Diet comprises of: carnivore (birds eating insects, frogs, small mammals), insectivore (birds eating strictly insects), and frugivore (birds eating fruits). Foraging methods include: foraging, which is plucking fruits (*S. caripensis*) or picking up food from branches or on the ground (*B. auritus, B. stellatus*), hawking (hunting on the wing), and sallying (leaping out from perches or the ground). Foraging heights cover low altitude (flying under the canopy), various altitude and high altitude (flying above the canopy). Habitat density consists of closed habitat (e.g. rainforest, woodlands), open habitat (e.g. open country, grassland, deserts, savannah), and semi-open (a mixture of open country and woodlands). Species names are colour-coded following their bristle morphotypes: frogmouths in purple, nighthawks and spotted nightjar in green, nightjars and pauraque in blue, Australian owlet-nightjar in brown, and oilbird in orange.

| **Species** | **Foraging time** | **Diet** | **Foraging method** | **Foraging heights** | **Habitat density** |
| --- | --- | --- | --- | --- | --- |
| **P. strigoides** | Crepuscular and nocturnal | Carnivore | Foraging | Low | Closed habitat |
| **B. auritus** | Nocturnal | Insectivore | Foraging | Low | Closed habitat |
| **B. stellatus** | Nocturnal | Insectivore | Foraging | Low | Closed habitat |
| **C. nacunda** | Crepuscular, nocturnal partially diurnal | Insectivore | Hawking | High | Open habitat |
| **C. minor** | Crepuscular and partially diurnal | Insectivore | Hawking | Various | Open habitat |
| **E. argus** | Crepuscular and nocturnal | Insectivore | Hawking and sallying | Low | Semi-open habitat |
| **C. pectoralis** | Crepuscular and nocturnal | Insectivore | Sallying | Various | Semi-open habitat |
| **C. vexillarius** | Crepuscular, nocturnal partially diurnal | Insectivore | Hawking | High | Semi-open habitat |
| **C. europaeus** | Crepuscular and nocturnal | Insectivore | Hawking | Various | Semi-open habitat |
| **N. albicollis** | Crepuscular and nocturnal | Insectivore | Sallying | Low | Closed habitat |
| **A. cristatus** | Crepuscular and nocturnal | Insectivore | Hawking and sallying | Various | Closed habitat |
| **S. caripensis** | Crepuscular and nocturnal | Frugivore | Foraging | Various | Closed habitat |

**Table S2. Morphotype groupings from the bristle morphology and follicle anatomy data**. Two k-means cluster analyses were conducted in Matlab (MATLAB and Statistics Toolbox Release 2019a, The MathWorks, Inc., Natick, Massachusetts, United States). The morphology variables included the discrete categories of bristle length, width, number and branching, and the anatomy variables included were the discrete categories of muscle bundle size, tissue density and Herbst corpuscle number. Both datasets were partitioned into five defined groups using k-means distance measures. The morphology grouping agreed with the phylogeny, but the follicle anatomy grouping did not, and varied from species to species.

| **Species** | **Bristle morphology morphotypes** | | **Follicle anatomy Morphotypes** | |
| --- | --- | --- | --- | --- |
| **P. strigoides** | **Group 1** | **Group 1** | |  |
| **B. auritus** | **Group 1** | | **Group 1** | |
| **B. stellatus** | **Group 1** | | **Group 1** | |
| **C. nacunda** | **Group 2** | | **Group 3** | |
| **C. minor** | **Group 2** | | **Group 3** | |
| **E. argus** | **Group 2** | | **Group 4** | |
| **C. pectoralis** | **Group 3** | | **Group 2** | |
| **C. vexillarius** | **Group 3** | | **Group 3** | |
| **C. europaeus** | **Group 3** | | **Group 5** | |
| **N. albicollis** | **Group 3** | | **Group 1** | |
| **A. cristatus** | **Group 5** | | **Group 2** | |
| **S. caripensis** | **Group 4** | | **Group 2** | |
